# Association between serum leucine-rich alpha-2-glycoprotein levels and characteristic enteroclysis findings in small intestinal Crohn’s disease

**DOI:** 10.1093/crocol/otag012

**Published:** 2026-02-18

**Authors:** Masayuki Fukata, Shu Kikuta, Kazuyo Okayama, Akira Sonoda, Soh Okano, Kiyohito Unuma

**Affiliations:** Center for Inflammatory Bowel Disease, Department of Internal Medicine, Tokyo Yamate Medical Center, Japan Community Healthcare Organization, Tokyo, Japan; Center for Inflammatory Bowel Disease, Department of Internal Medicine, Tokyo Yamate Medical Center, Japan Community Healthcare Organization, Tokyo, Japan; Center for Inflammatory Bowel Disease, Department of Internal Medicine, Tokyo Yamate Medical Center, Japan Community Healthcare Organization, Tokyo, Japan; Center for Inflammatory Bowel Disease, Department of Internal Medicine, Tokyo Yamate Medical Center, Japan Community Healthcare Organization, Tokyo, Japan; Center for Inflammatory Bowel Disease, Department of Internal Medicine, Tokyo Yamate Medical Center, Japan Community Healthcare Organization, Tokyo, Japan; Department of Radiology, Saitama North Medical Center, Japan Community Healthcare Organization, Saitama, Japan

**Keywords:** Crohn’s disease, LRG, small intestine, enteroclysis

## Abstract

**Introduction:**

Enteroclysis provides a whole picture of mucosal and wall-structural information that is advantageous in assessing small intestinal Crohn’s disease (CD). Leucine-rich alpha-2-glycoprotein (LRG) is an acute-phase protein that has been shown to be increased in active CD. We sought to explore the association of LRG with the presence of typical CD findings in enteroclysis.

**Methods:**

Patients with small intestinal CD whose serum LRG and C-reactive protein (CRP) were measured within 30 days before or after enteroclysis were selected, and were categorized by the presence of longitudinal ulcer, cobblestone appearance, and stricture/narrowing. Levels of LRG were compared by the type and the extent of lesions. Detection performances of LRG and CRP were compared for the presence of each type of lesion.

**Results:**

Serum LRG levels were significantly higher in patients who had enteroclysis findings than patients with no findings (23.5 ± 9.2 vs. 12.8 ± 3.0 μg/dL, *P *< .00001). The level of LRG was not affected by disease extent or history of bowel resection. LRG over 16.3 μg/dL had good detection accuracy for the presence of CD lesions with an area under the receiver operating characteristic curve of 0.85 (95% confidence interval: 0.75-0.95). The association of LRG with the presence of CD-specific lesions was significantly higher than CRP, especially for the presence of stricture/narrowing (AUC: 0.82 vs. 0.67, *P *= .005) and longitudinal ulcer (AUC: 0.93 vs. 0.80, *P *= .017).

**Conclusion:**

Elevation of serum LRG is associated with the presence of typical CD lesions in the small intestine that may be found in entericlysis.

## Graphical Abstract

**Graphical Abstract:**
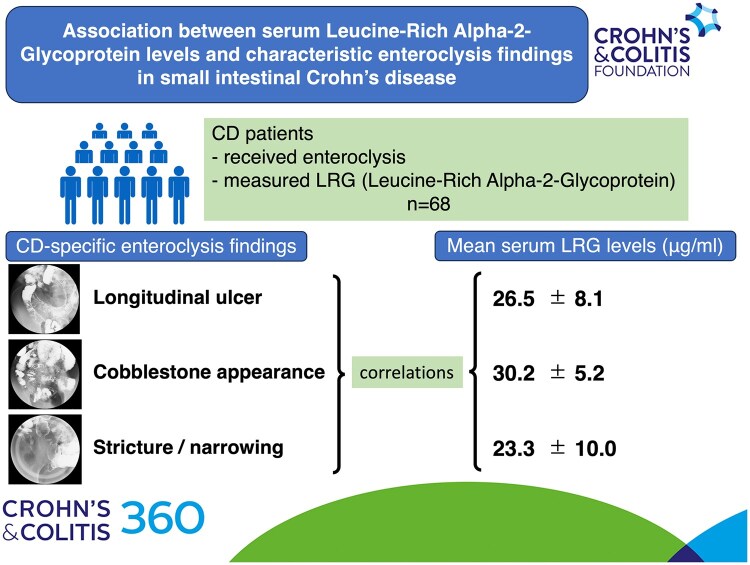

## Introduction

Crohn’s disease (CD) is a chronic inflammatory disorder which may affect any part of the gastrointestinal tract. The characteristics of CD lesions in diagnostic imaging include transmural inflammation and longitudinal deep ulcers that tend to be involved in the mesenteric side of the tract with discrete distribution. Cobblestone appearance, made up of intestinal mucosa cubically divided by multiple deep ulcers, has been thought to be a typical radiological or endoscopic finding of CD and thus it is useful in making diagnosis.^1^

Chronic transmural inflammation of the intestine in patients with CD may cause severe deformity of the intestinal tract by forming strictures and fistulas that require surgical treatment. The life-long risk of bowel resection in patients with CD has been around 44%-50%.^2^^,^^3^ Earlier intervention with biologics before a deformity of the intestinal tract develops has been expected to modify the disease course of patients with CD.^4^^,^^5^ In order to treat patients with CD before an intestinal deformity develops, a practical screening method to detect early CD lesions in the small intestine is needed, although the detection of early CD lesions in the small intestine is often difficult. Recently, small intestinal endoscopy has been used to screen small intestinal lesions in patients with CD, but it can be invasive and difficult to examine entire small intestine with a unidirectional approach. The cross-sectional imaging studies, such as computed tomography enterography (CTE) or magnetic resonance enterography (MRE), are noninvasive. However, detection of mucosal ulcers and making a diagnosis of CD by differentiating it from other inflammatory diseases may be difficult by those cross-sectional studies alone.^6^^,^^7^ On the other hand, enteroclysis is advantageous in assessing the wall structure in detail and localizing the lesions within a picture of the whole small intestine.

Recently, several biomarkers have been applied to predict disease status of CD.^8^ Leucine-rich alpha-2-glycoprotein (LRG) is an acute phase protein that can be expressed by hepatocytes and local neutrophils, endothelial cells, and epithelial cells at the site of inflammation.^9^ Several reports have shown increased serum levels of LRG in active CD that can be decreased by treatments along with the reduction of disease activity.^10–13^ The increased serum levels of LRG can be seen in asymptomatic patients with CD who show ulcerations in the small intestine in endoscopic examination.^14^ In this study, we examined whether serum levels of LRG correlate with the presence of each type of CD lesion typically found in enteroclysis. Identifying a better biomarker that indicates the presence of typical CD lesions in the small intestine will improve the management of CD in clinical practice that may contribute to a better prognosis for each patient.

## Methods

### Subjects and data collection

We conducted a retrospective observational study. By searching for “Crohn’s disease” in the diagnosis entry system of the electronic medical record within the enteroclysis registry of the radiology department, there were 1179 consecutive patients with CD who received enteroclysis between June 2020 and October 2021. The diagnosis of CD was confirmed based on the clinical manifestations, endoscopic findings, pathology results of mucosal biopsy or resected bowel specimens, and clinical course of the disease with reference to the practice guidelines.^15^ LRG and C-reactive protein (CRP) were routinely measured in our clinical laboratory using commercial assay kits (Sekisui Medical Co., LTD). Based on the electronic records of the endoscopic and radiological studies, disease phenotype was defined according to the Montreal classification^16^ and only patients who were confirmed to have no active CD lesions in any parts of the gastrointestinal tract besides the small intestine were selected. Patients with active perianal disease and extra intestinal manifestations were also excluded. In this study, 3 findings of enteroclysis were selected as typical CD lesions^1^; longitudinal ulcer,^2^ cobblestone appearance, and^3^ stricture/narrowing, and the cases overlapping these 3 findings were documented.

Patient demographics and characteristics of the disease including duration of illness, sex, disease behavior, history of bowel resection, and results of blood tests were manually obtained from the electronic medical record. Serum LRG and CRP levels measured on the same day within 30 days before or after enteroclysis were used to compare to each finding of the small intestine.

### Enteroclysis

**Figure 1:**
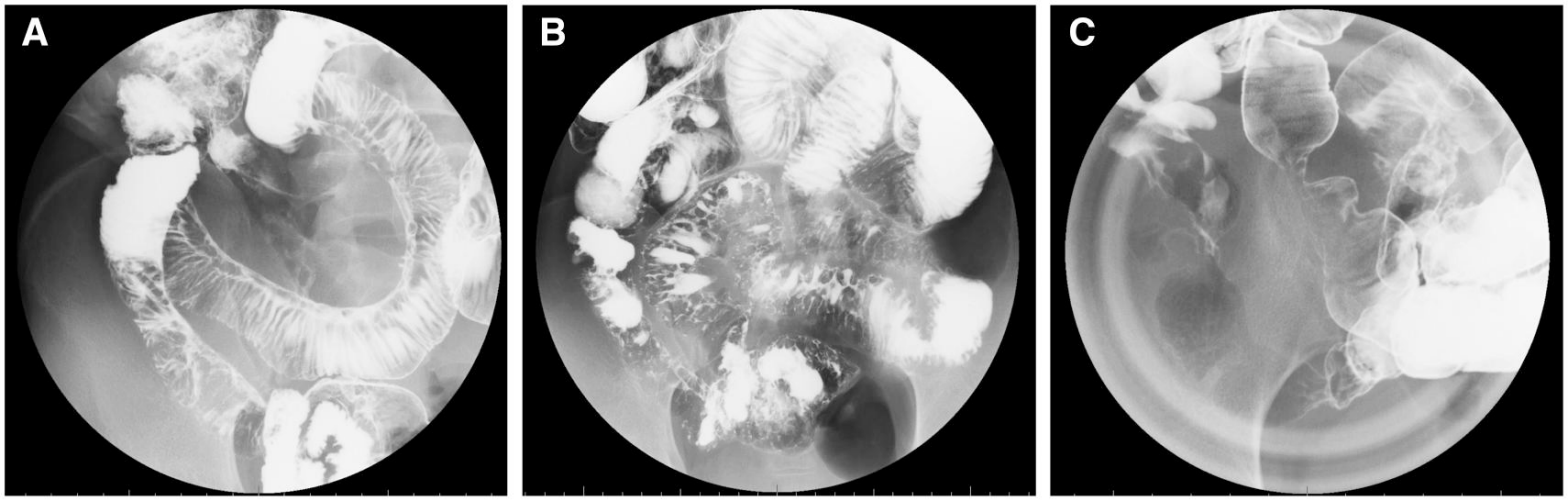
Representative entericlysis images of (A) longitudinal ulcer, (B) cobblestone appearance, and (C) stricture/narrowing.

Enteroclysis was performed by the method described previously.^17^ Briefly, a 10Fr catheter (Argyle, CardinalHealth) was introduced to the nasal cavity passing through the esophagus and the stomach, and the tip of the tube was placed on the proximal jejunum under fluoroscopic guide. After inflating the balloon with 15-20 mL of air, 150-300 mL of aqueous barium sulfate (Barytgen-Deluxe, Fushimi Laboratory) was injected through the catheter with a 50 mL syringe until the barium reached the distal small intestine. An adequate amount of air was then introduced to obtain the double contrast images. The longitudinal ulcer was identified as a vertically extended line along with the intestinal tract, which typically created a firm bow-shaped deformity (Figure 1A). The cobblestone appearance, a highly specific finding of CD, was identified as a nodular mucosal area that looked like cobblestone (Figure 1B). The stricture/narrowing was defined as an obviously narrow segment that was not affected by peristalsis (Figure 1C). Results were routinely reviewed by attending physicians and the diagnostic reports were made with the assistance of experienced technicians in the radiology department. The enteroclysis images of each patient were further reviewed by 2 gastroenterologists for this study.

### Statistical analysis

Categorical variables are summarized as frequencies and percentages, and continuous variables are expressed as means ± standard deviations (SD). Sample distributions are confirmed by comparison between medians and interquartile ranges. Percentages were compared by Fisher’s exact probability test or Chi-square test for the comparisons of disease phenotypes. The Mann–Whitney’s *U* test was used to compare differences between 2 independent variables, and multiple comparisons were performed with Kruskal–Wallis test for 3 or more variables using GraphPad Prism 8.0 (GraphPad Software Inc.). The receiver operating characteristic (ROC) analysis was performed to assess overall diagnostic performance of LRG and CRP for positive findings of CD lesions in enteroclysis and DeLong’s test was performed to compare area under receiver operating characteristic curves (AUROCs) by using EZR 4.0.2 (The R Foundation). Multivariate logistic regression was used to estimate the association of LRG, age, gender and the history of bowel resection with the probability of positive enteroclysis findings. A cut-off value was set to determine the presence or absence of CD lesions. *P*-values of less than .05 were considered to be significant.

### Ethical considerations

The institutional review board (IRB) approved the protocol (J-162) and the permission to use the medical records in each patient was obtained by opt-out.

## Results

### Patient demographics and characteristics

**Figure 2:**
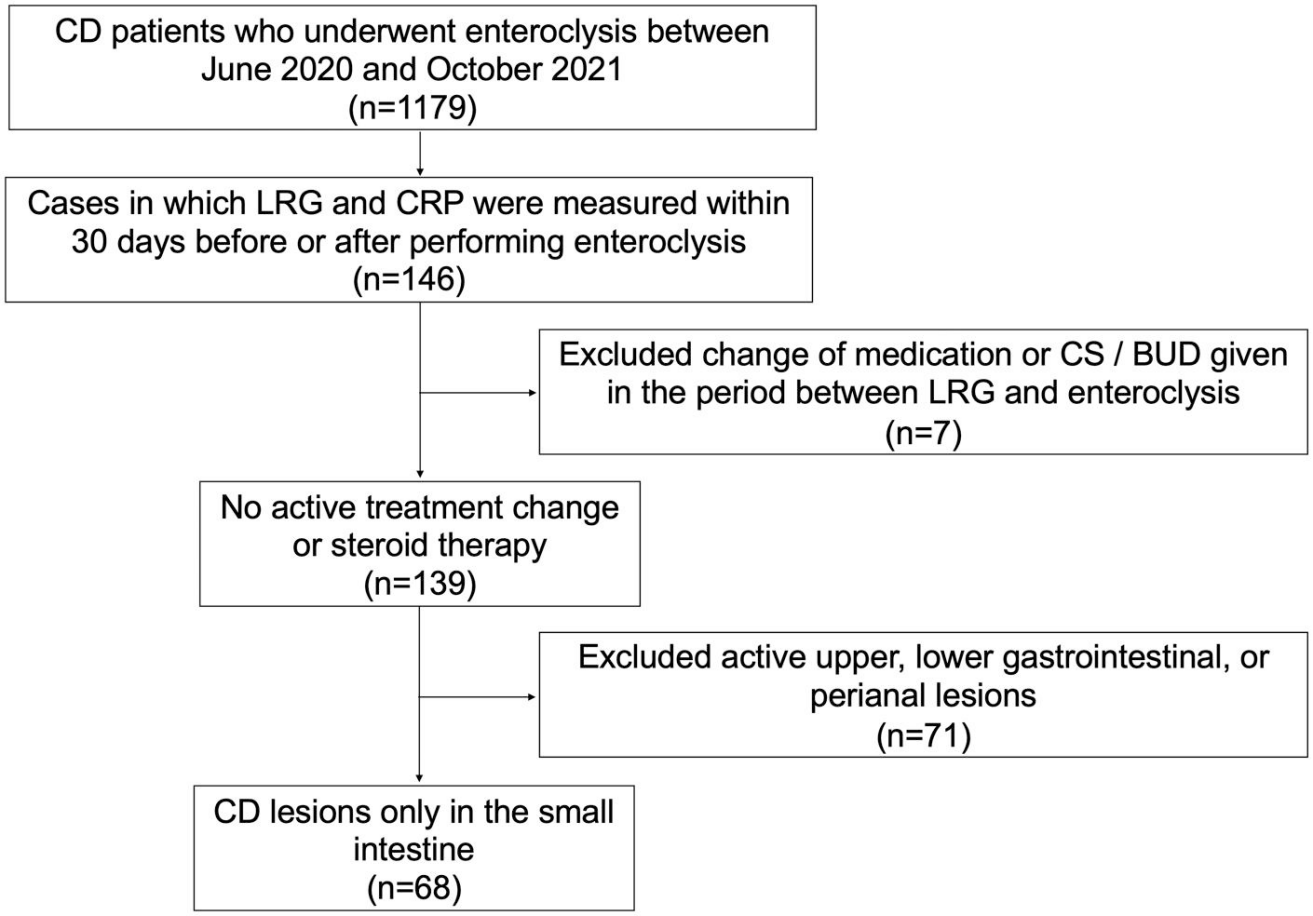
Flow diagram of patient selection. BUD, budesonide; CS, corticosteroid.

Among 1179 patients with CD, 146 patients whose LRG and CRP had been measured within 30 days before or after the enteroclysis were identified (Figure 2). LRG and CRP were measured on the same day in all cases and the mean difference between the serum assay and enteroclysis was 11.0 ± 10.3 days. Seven cases were excluded because their medications were changed or corticosteroid (CS) or budesonide was initiated during the period between enteroclysis and the serum assay. Cases that had active CD lesions besides the small intestine, minimal lesions such as aphthous ulcers, fluffy mucosa, and lesions with uncertain reproducibility were also excluded. Finally, a cohort of 68 cases were enrolled for the analysis.

Patient characteristics are shown in Table 1. Mean age and disease duration of the patients were 43.7 ± 11.1 and 19.0 ± 10.1 years, respectively, and 80.9% were male. All patients were Asians. There were 13 (19.1%) non–stricturing and non–penetrating, 36 (52.9%) stricturing, 10 (14.7%) penetrating, and 9 (13.2%) stricturing and penetrating types, and 30 patients (44.1%) had history of small bowel resection. Almost half of the patients (47.1%) were small intestinal type (L1), but there were 36 patients (52.9%) who were originally diagnosed as L3 type. Among the patients with L3 type, 18 patients (50.0%) had undergone total proctocolectomy and ileostomy, and 3 patients (8.3%) with sub-total colectomy with ileo-rectal or ileo-sigmoid anastomosis had no active colonic lesions confirmed by colonoscopy within 2 months of the LRG measurement. The remaining 15 patients (41.7%) comprised of 4 patients who initially had ileocecal CD lesions but have long been detected only small intestinal lesions after ileocecal resection, and 11 patients who had been confirmed normal colonic mucosa by colonoscopy within 2 months of the LRG measurement. All L2 patients were excluded from this study. Most patients (79.4%) were taking 5-aminosalicylic acid (5-ASA). Thioprine was taken 29.4% of the patients, and biological agents including anti-TNF-α, Ustekinumab, and Vedolizumab, were used in 20.6% of the cases. The mean value of LRG was 17.7 ± 8.5 μg/mL. Positive enteroclysis findings were seen in 32 (47.1%) patients (Table 2). There were no differences in demographics between patients with or without positive enteroclysis findings. Only the disease behavior was different between patients with or without positive findings, mainly because more patients with negative findings were B1 phenotype (30.6%) than patients with positive findings (6.3%). We then divided patients into LRG positive and negative with the commercial cut off value of 16 μg/mL (Sekisui Medical Co., LTD).^18^ There were 41 LRG-positive and 27 LRG-negative patients (Table 3). Age, gender, disease duration, and the rate of past bowel resection were similar between them, but LRG positivity was different based on the disease behavior and there were more patients with B1 behavior in LRG-positive than LRG-negative individuals.

**Table 1:** Characteristics of patients.

Small intestinal Crohn’s disease (CD) patients （*n* = 68）	
**Age (±SD)**	43.7 ± 11.1
**Sex (male %)**	55 (80.9%)
**Duration of illness (years** ± **SD)**	19.0 ± 10.1
**Disease behavior (%)**	
** Non–stricturing, non–penetrating (B1)**	13 (19.1%)
** Stricturing (B2)**	36 (52.9%)
** Penetrating (B3)**	10 (14.7%)
** Stricturing and penetrating (B2+B3)**	9 (13.2%)
** Ileal (L1)**	32 (47.1%)
** Colonic (L2)**	0 (0.0%)
** Ileocolonic (L3)**	36 (52.9%)
**History of small bowel resection**	30 (44.1%)
**Medications**	
** 5-aminosalicylic acid**	54 (79.4%)
** Thioprine**	20 (29.4%)
** Anti-TNF-a**	11 (16.2%)
**Ustekinumab (UST)**	2 (2.9%)
** Vedolizumab (VDZ)**	1 (1.5%)
**Positive enteroclysis finding**	32 (47.1%)
**Leucine-rich alpha-2-glycoprotein (LRG) (±SD, μg/mL)**	17.7 ± 8.5

**Table 2:** Demographics of patients with positive and negative findings in enteroclysis.

Patients （*n* = 68）	Positive *n* = 32	Negative *n* = 36	*P*
**Age （± SD）**	44.4 ± 11.0	43.0 ± 11.3	.70
**Sex (male %)**	27 (%)	28 (%)	.55
**Duration of illness (years** ± **SD)**	20.8 ± 9.2	17.7 ± 10.6	.27
** Non–stricturing, non–penetrating (B1)**	2 (6.3%)	11 (30.6%)	<.01
** Stricturing (B2)**	21 (65.6%)	15 (41.7%)	
** Penetrating (B3)**	4 (12.5%)	6 (16.7%)	
** Stricturing and penetrating (B2+B3)**	5 (15.6%)	4 (11.1%)	
** Ileal (L1)**	17 (53.1%)	15 (41.7%)	.47
** Ileocolonic (L3)**	15 (46.9%)	21 (58.3%)	
**History of bowel resection (%)**	15 (46.9%)	15 (41.9%)	.81

**Table 3:** Demographics of Leucine-rich alpha-2-glycoprotein (LRG) positive and negative patients (cut off ≥16 μg/mL).

Patients （*n* = 68）	LRG positive *n* = 41	LRG negative *n* = 27	*P*
**Age （± SD）**	42.6 ± 10.6	45.3 ± 11.9	.37
**Sex (male %)**	31 (75.6%)	24 (88.9%)	.22
**Duration of illness (years ± SD)**	17.4 ± 9.6	21.8 ± 10.4	.07
** Non–stricturing, non–penetrating (B1)**	12 (29.3%)	1 (3.7%)	<.01
** Stricturing (B2)**	20 (48.8%)	16 (59.3%)	
** Penetrating (B3)**	6 (14.6%)	4 (14.8%)	
** Stricturing and penetrating (B2+B3)**	3 (7.3%)	6 (22.2%)	
** Ileal (L1)**	18 (43.9%)	13 (48.1%)	.62
** Ileocolonic (L3)**	23 (56.1%)	13 (48.1%)	
**History of bowel resection (%)**	12 (29.3%)	18 (66.7%)	.79

### LRG was elevated in patients with specific enteroclysis findings of CD in the small intestine

There were 36 patients (52.9%) who had no positive findings in the enteroclysis, but 32 patients (47.1%) showed typical CD lesions including longitudinal ulcer (46.9%), cobblestone appearance (15.6%), and stricture/narrowing (75.0%) (Table 4). All patients with cobblestone appearance had an overlap finding of longitudinal ulcer, and 2 of them also had stricture/narrowing. There were 7 patients who showed both longitudinal ulcer and stricture/narrowing. The average serum LRG level of patients who had positive findings in the enteroclysis was 23.5 ± 9.2 μg/dL, which was significantly higher than the LRG levels in patients without positive findings (12.8 ± 3.0 μg/dL, *P *< .00001). Multivariate analysis including age, gender and the history of bowel resection confirmed the serum LRG level as an independent factor for the presence of positive findings in enteroclysis (odds ratio [OR] 1.4, 95% confidence interval [CI]: 1.2-1.6, *P *= .0001). In addition, The LRG levels of patients with each finding were individually higher when compared with the LRG levels of patients without positive findings. Therefore, the elevation of serum LRG may be highly associated with the presence of a positive finding in enteroclysis in patients with small intestinal CD.

**Table 4:** Leucine-rich alpha-2-glycoprotein (LRG) levels of patients in each finding of enteroclysis.

Enteroclysis results	No of patients (%)	LRG (μg/ml)	*P* ^a^
**No findings**	36 (52.9)	12.8 ± 3.0	NA
**Positive findings**	32 (47.1)	23.5 ± 9.2	<.00001
** Stricture/Narrowing**	24 (75.0% of findings)	23.3 ± 10.0	<.0001
** Longitudinal ulcer**	15 (46.9% of findings)	26.5 ± 8.1	<.00001
** Cobblestone appearance**	5 (15.6% of findings)	30.2 ± 5.2	<.0001

a Compared with no findings.

### Serum LRG levels and areas of the small intestine affected by CD lesions

**Figure 3:**
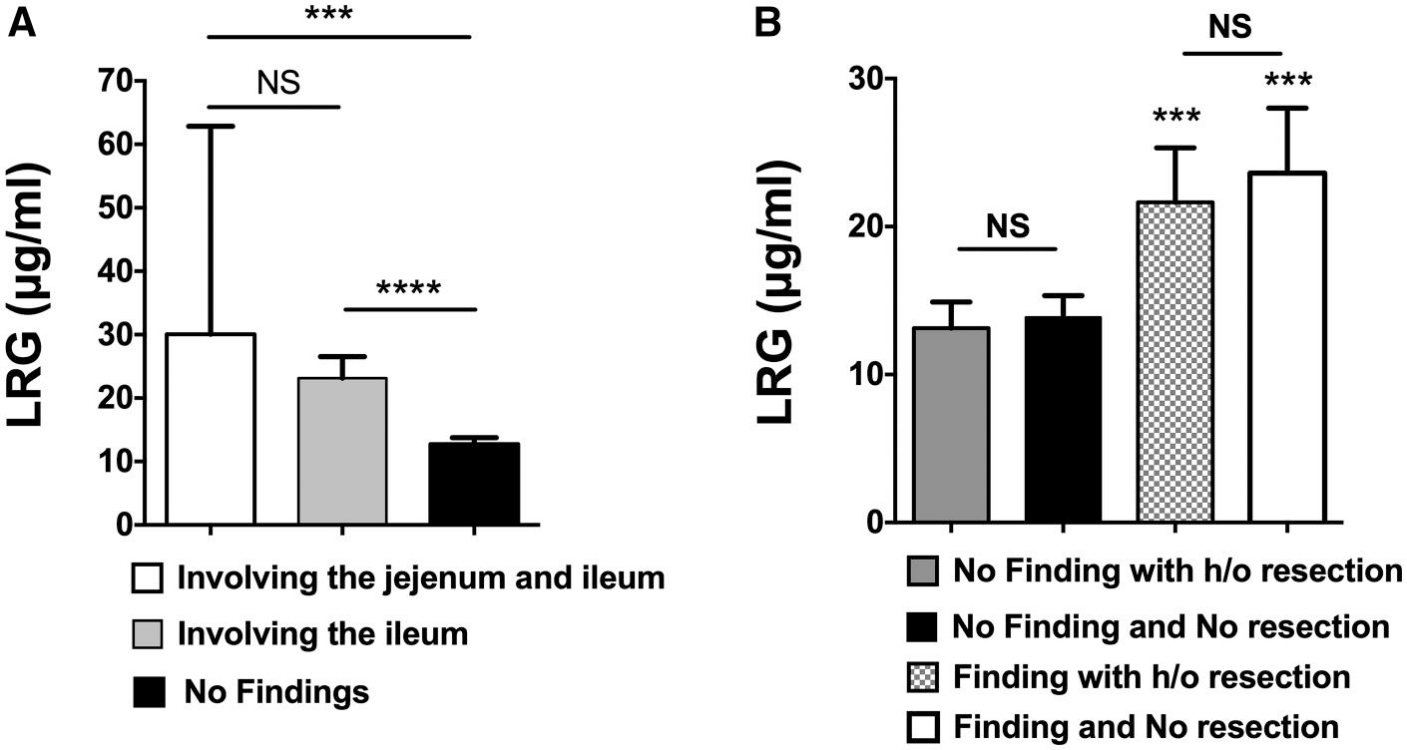
Changes of serum leucine-rich alpha-2-glycoprotein (LRG) levels with disease distribution and history of bowel resection. (A) Comparison of serum LRG levels based on the disease affected area of the small intestine. ***P < .001, ****P < .0001 (B) Comparison of LRG levels with or without history of small bowel resection. NS: not significant.

While we wanted to examine the correlation between the LRG levels and the areas of CD lesions involved in the small intestine, it was difficult to measure the extent of CD lesions. Therefore, we compared the LRG levels of patients who had CD lesions only in the ileum, expanded jejunum to ileum, and patients with no CD lesions (Figure 3A). Although the LRG levels in patients with extensive disease were relatively higher than that in patients with CD lesions limited in the ileum, the difference in their LRG levels was not statistically significant and the LRG levels in both groups were individually higher than the LRG levels in patients with no CD lesions.

We concerned that the history of bowel resection might influence the relationship between serum LRG levels and the results of enteroclysis. We then stratified the comparison data by the history of small bowel resection (Figure 3B). There was no difference in serum LRG levels between the patients with history of small bowel resection and those with no history of resection (18.0 ± 9.0 μg/dL vs. 17.7 ± 8.3 μg/dL, *P *= .64). Multiple comparison analyses demonstrated significantly higher LRG levels in patients with positive findings in the enteroclysis regardless of past history of bowel resections.

### Elevated serum LRG is associated with the presence of CD-specific findings in the small intestine

**Figure 4:**
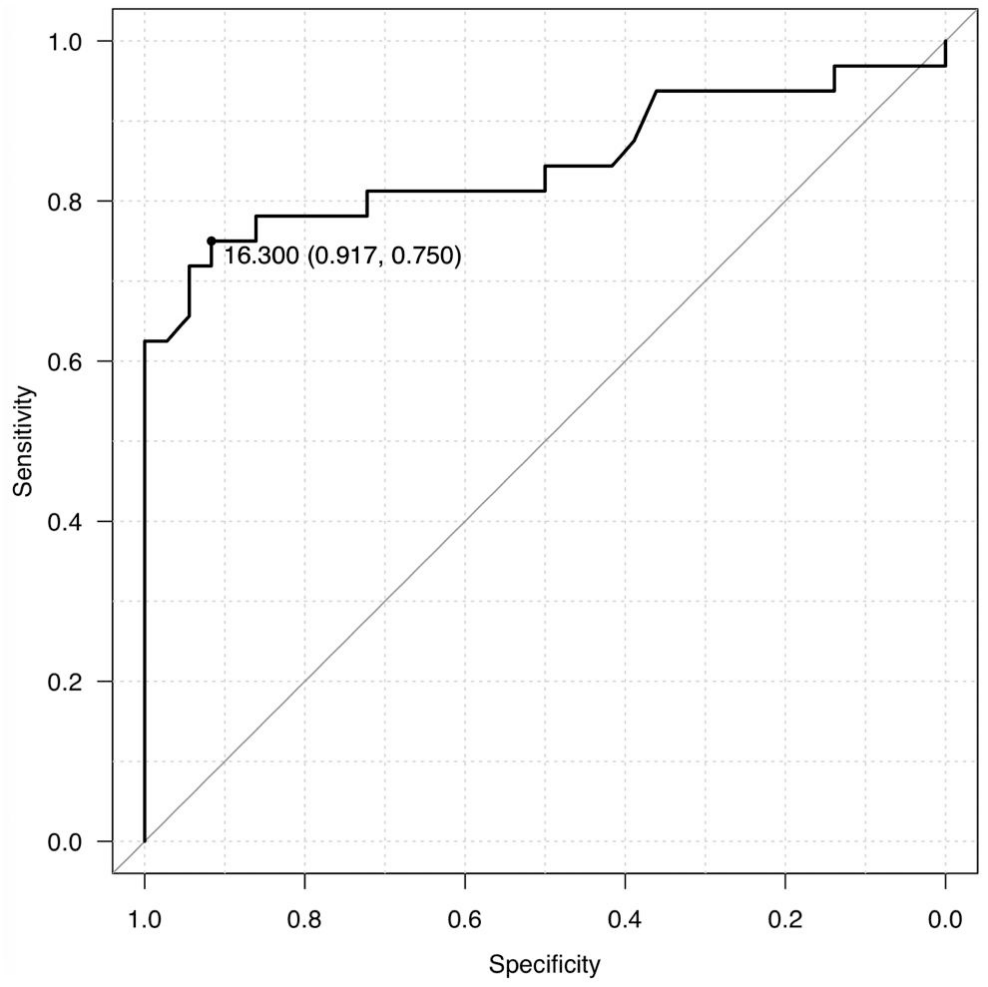
Receiver operating characteristic (ROC) curve for leucine-rich alpha-2-glycoprotein (LRG) to predict the presence of Crohn’s disease (CD) lesions in enteroclysis.

Next, we assessed the performance of LRG as a biomarker to estimate positive findings of each type of CD lesion in enteroclysis. LRG had good performance for the estimation of the presence of any CD lesions with an area under the ROC curve of 0.85 (95% CI, 0.75-0.95), and the best cut-off value was 16.3 μg/dL with sensitivity of 0.75 and specificity of 0.92 (Figure 4). When we used the cut-off value of LRG 16.3 μg/dL for the probability of existence in each type of CD lesion, the sensitivity and negative predictive value were highest in cobblestone appearance among 3 CD findings (Table 5). In addition, the sensitivity and negative predictive value were higher in longitudinal ulcer relative to stricture/narrowing. Therefore, LRG of less than 16.3 μg/dL can be at least an indication to exclude possibilities of having longitudinal ulcers and/or cobblestone appearances in patients with small intestinal CD.

**Table 5:** The performance of Leucine-rich alpha-2-glycoprotein (LRG) > 16.3 μg/dL for predicting each Crohn’s disease (CD) finding in enteroclysis.

Enteroclysis results	Sensitivity (%, 95% confidence interval [CI])	Specificity (%, 95% CI)	Positive predictive value (PPV) (%, 95% CI)	Negative predictive value (NPV) (%, 95% CI)	*P*
**Strictures/Narrowings**	68.2 (45.1-86.1)	91.7 (77.5-98.3)	83.3 (58.6-96.4)	82.5 (67.2-92.7)	<.0001
**Longitudinal ulcers**	93.3 (68.1-99.8)	91.7 (77.5-98.3)	82.4 (56.6-96.2)	97.1 (84.7-99.9)	<.0001
**Cobblestone appearance**	100 (47.8-100.0)	91.7 (77.5-98.3)	62.5 (24.5-91.5)	100 (89.4-100.0)	<.0001

### LRG shows greater associations to the presence of each CD lesion than CRP

**Figure 5:**
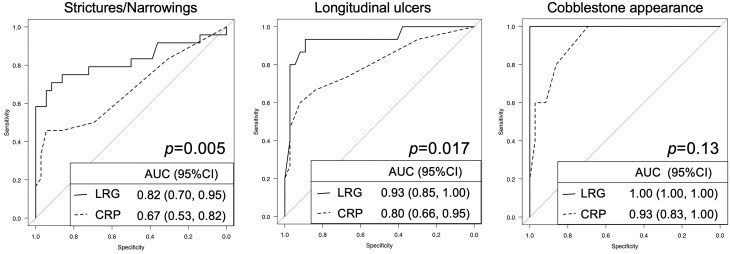
Receiver operating characteristic (ROC) curves of subgroup analysis. Performances of leucine-rich alpha-2-glycoprotein (LRG) and C-reactive protein (CRP) are compared for the prediction of each CD finding in enteroclysis, stricture/narrowing (*n* = 24), longitudinal ulcer (*n* = 15), cobblestone appearance (*n* = 5).

CRP has long been used to monitor the disease activity of CD regardless of the location and the type of lesions. We therefore compared LRG and CRP in their efficacy of estimating the presence of each type of small intestinal CD lesion. In our study, CRP of more than 0.4 mg/dL was associated with the presence of small intestinal lesions with an area under the ROC curve of 0.71 (95% CI, 0.59-0.83) and the sensitivity and specificity of 0.53 and 0.82, respectively. There was a statistically significant difference in AUROCs between LRG and CRP for the estimation of positive enteroclysis findings (*P *= .024). In the ROC analysis of individual types of CD lesion, LRG levels more than 16.3 μg/dL demonstrated significantly higher performance for estimating the presence of each type of CD lesion than CRP (the cut-off values of CRP that estimate as highest performance was 0.5 mg/dL in stricture/narrowing, and 0.5 mg/dL in longitudinal ulcer), although the difference was not statistically significant in the detection of cobblestone appearances (highest performance cut-off in LRG; 26.4 μg/dL and in CRP; 0.2 mg/dL) (Figure 5).

## Discussion

Initial presentation of CD is often elusive and can be just abdominal pain or unintentional weight loss. In addition, a screening colonoscopy and an esophagogastroduodenoscopy (EGD) may not lead to the diagnosis of CD if it is jejuno-ileitis type. The sequelae of unrecognized jejuno-ileal CD is often associated with strictures and/or fistulas that are difficult to treat and may require surgical resection of the affected intestine.^19^ It has been suggested that therapeutic escalation based on the sequential disease monitoring and achieving the goal in an appropriate time period, called “treat-to-target,” may improve the long-term prognosis of CD.^20^ Therefore, timely diagnosis and monitoring of disease activities are crucial for a better prognosis in patients with small intestinal CD. Recently, several imaging modalities have been applied to assess small intestinal CD lesions but most of them require special equipment or skills to complete. Therefore, a more accessible method to screen for small intestinal CD would be needed to make an earlier diagnosis before an irreversible wall deformity develops in the small intestine. In this study, we showed that the elevation of serum LRG levels was associated with the presence of typical CD lesions identified in enteroclysis in patients with small intestinal CD. Measurements of LRG in patients with small intestinal CD that presented no lesions in EGD and colonoscopy may be used to select candidates for further examination of the small intestine.

There have been attempts to monitor the disease activity of CD with biomarkers. CRP is the most utilized serum biomarker in clinical practice that is easily measurable but is produced mainly by IL-6 in the liver regardless of the cause of its expression. Recent studies have shown that LRG can be used as a biomarker to assess the disease activity in inflammatory diseases, including CD.^21^ LRG was isolated from human serum in 1977 as a glycoprotein belonging to the alpha2-globulins,^22^ and later found to be induced not only by IL-6 but also by other cytokines including TNF-α, IL-1β, and IL-22, all known to be involved in the pathogenesis of CD.^9^ Recently, a correlation between the serum LRG levels and the endoscopic severity of CD has been reported.^23^^,^^24^ Detection of CD lesions by other imaging modalities, including MRE, CTE, and intestinal ultrasound, has also been found to be associated with LRG elevation, and the cut-off value of LRG that predicts the presence of a positive finding in these modalities ranged from 14 to 16 μg/dL.^12^^,^^18^^,^^25^ However, the correlation and the extent of LRG elevation with CD-specific findings of enteroclysis have not been explored. Enteroclysis has not been utilized as it used to be and its quality depends on the skill of the operator. Yet some small intestinal CD can be diagnosed only by enteroclysis, especially in cases with stenosis and/or fistulas. For example, small bowel endoscopy may have limitations in passing stenosis and diagnosing fistulas. In addition, stenotic lesions without wall thickness or dilation of the proximal loop, fissuring ulcers, and mucosal ulcers are often difficult to detect in cross-sectional imaging studies.^7^^,^^26^ While intestinal ultrasound has been used as a minimally invasive cross-sectional imaging test for IBD, there are still some difficulties for its routine use, such as its low screening sensitivity.^6^ Using these cross-sectional imaging studies might make it difficult to identify characteristic lesions of CD, such as cobblestone appearances, and thus also make it difficult to have a definite diagnosis by distinguishing CD from other small intestinal diseases. Enteroclysis is more advantageous than other modalities as it can show an image of the whole small intestine with detailed mucosal relief patterns regardless of the presence of stenosis or wall thickness. Although previous studies have shown the usefulness of LRG in predicting mucosal healing in the small intestine by comparing with endoscopic or other imaging findings besides enteroclysis,^18^^,^^27–29^ our study demonstrates the correlation of serum LRG levels with the presence of CD-specific enteroclysis findings. Therefore, we believe that this study has potential implications for future research on LRG and its correlation with other modalities in the more precise assessment of small intestinal lesions in patients with CD. Identifying LRG as a clinically useful biomarker that indicates the possible existence of CD-specific lesions will help in selecting patients who are candidates for enteroclysis or other imaging studies to make a diagnosis and monitor the disease course of CD.

The correlation with LRG was more pronounced in the presence of longitudinal ulcers and cobblestone appearances than in stricture or narrowing. This can be due to the heterogeneous causes of stenotic lesions that involve individually different degrees of inflammatory and fibrotic components. Although an intestinal stenosis itself has been implicated with serum LRG elevation, there might be a limitation in detecting and monitoring the stenotic CD lesions with the changes of serum LRG levels.^23^ Our study shows that the cobblestone appearances, the most sensitive and specific findings for CD, were highly correlated with LRG. While our study demonstrates a significant correlation of serum LRG elevation with the presence of CD-specific lesions detected by enteroclysis, there are some limitations. Because the utilization of enteroclysis has decreased, the direct generalizability of these findings might be limited. The retrospective data collection of LRG, which does not measure all the patients who are undergoing enteroclysis, might induce selection biases. About half of the patients were treated only with 5-ASA (45.6%) in this study, which may limit the generalizability of the data and potentially represent a source of selection bias, given that the lack of efficacy of 5-ASA in CD is known. Although our results did not show an association between the extent of disease and the levels of LRG, the degree of LRG elevation may depend on the severity of inflammation composed not only of the area affected but also of the depth of disease in small intestinal CD. In any case, further research would be warranted to determine the pathological mechanisms underlying LRG production from different morphological types of mucosal lesions in patients with CD.

## Conclusion

The measurement of LRG would be useful to indicate the existence of small intestinal CD lesions that can be detected by enteroclysis. Since LRG showed stronger association with some of the CD-specific lesions than CRP, the long-term prognosis of newly diagnosed CD, especially the jejuno-ileitis type, may be improved with the disease monitoring by LRG.

## Data Availability

The data that support the findings of this study are available from the corresponding author upon reasonable request.
